# A 12-year expanding pink plaque on the chest

**DOI:** 10.1016/j.jdcr.2025.11.058

**Published:** 2026-01-22

**Authors:** Farshid Etaee, Frank Castiglione, Xingyuan Jiang, Ronghua Hu, Anjela Galan, Keith A. Choate, Matthew D. Vesely

**Affiliations:** a Department of Medicine, Yale University, New Haven, Connecticut; b Dermatology Physicians of Connecticut, Hamden, Connecticut; c Department of Dermatology, Yale University, New Haven, Connecticut; d Department of Pathology, Yale University, New Haven, Connecticut; e Department of Genetics, Yale University, New Haven, Connecticut

**Keywords:** cholesterol, lovastatin, porokeratosis

## Case

**Fig 1:**
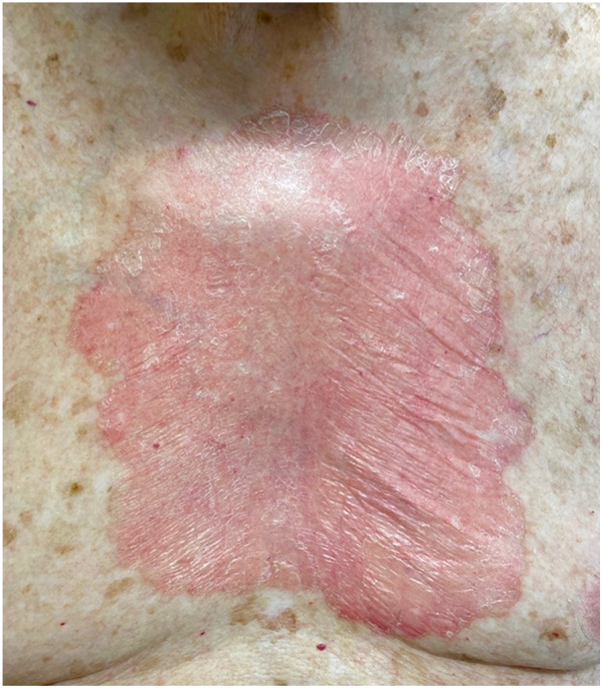
Clinical image. Clinical appearance of plaque.

**Fig 2:**
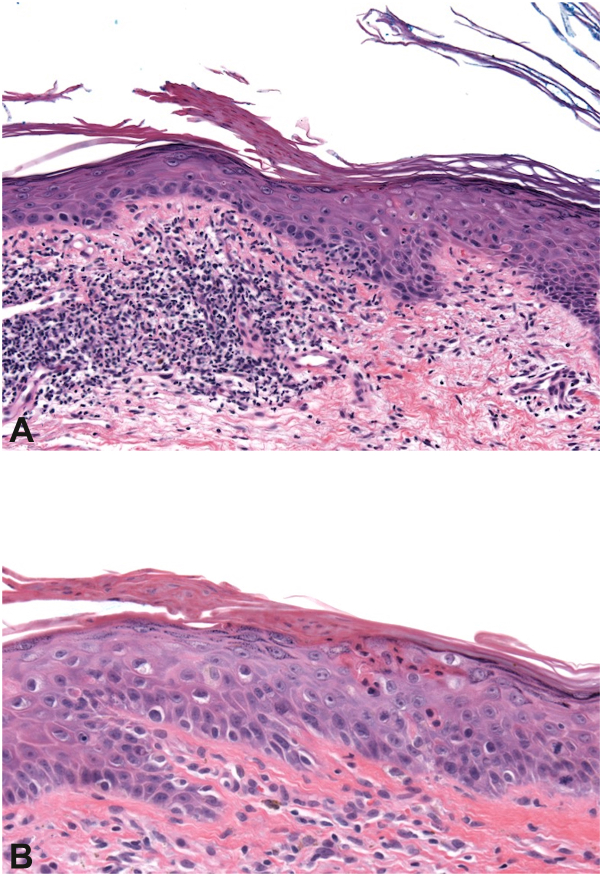
Histopathology of biopsies. Histological evaluation of 2 different biopsies at different timepoints from the same lesion **(A** and **B)** showed cornoid lamella, dyskeratotic keratinocytes and superficial lymphohistiocytic inflammation.

**Fig 3:**
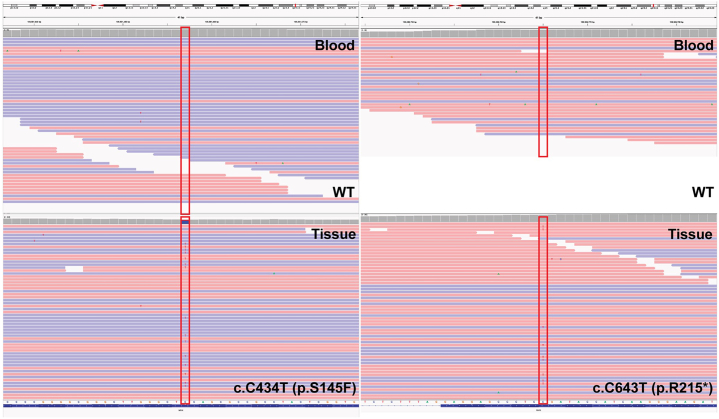
Somatic *MVK* mutations detected in affected tissue but absent in blood. Integrative Genomics Viewer (IGV) visualization of whole-exome sequencing reads. The heterozygous *MVK* variants c.C434T (p.S145F) (*left*) and c.C643T (p.R215∗) (*right*) were present in affected tissue (*bottom panels*) but absent in matched blood DNA (*top panels*). Reads are colored by strand: *blue*, forward strand; *red*, reverse strand.

A 95-year-old man presented with an expanding plaque on the chest over a 12-year period. Skin examination showed 21 × 15 cm pink, atrophic, scaly plaque with elevated pink-red border on his central chest (Fig 1). The patient was most bothered by the associated itch. Over a decade, multiple biopsies were performed and all showed cornoid lamella, dyskeratotic keratinocytes, and superficial lymphohistiocytic inflammation (Fig 2). Whole-exome sequencing performed on blood and affected tissue identified 2 somatic *MVK* (mevalonate kinase) mutations present exclusively in affected tissue (Fig 3).

**Question: What is the diagnosis?**

**A.** Marjolin ulcer
**B.** Bowen disease
**C.** Giant porokeratosis
**D.** Majocchi granuloma
**E.** Lues maligna

**Answer: Giant porokeratosis**

## Discussion

Porokeratosis is a disease of keratinization due to inherited or acquired mutations of keratinocytes within the mevalonate pathway.^1^ There are multiple clinical variants of porokeratosis, including classical porokeratosis of Mibelli, linear porokeratosis, disseminated superficial actinic porokeratosis, and porokeratosis palmaris plantaris et disseminatum. All variants of porokeratosis share mutations in the mevalonate pathway and histopathologically show cornoid lamellae. Giant porokeratosis is considered a morphological variant of porokeratosis of Mibelli that expands to a very large size, as in our patient.

Porokeratosis is recognized as a premalignant disease with a malignant transformation incidence of 7.5%; thus, early diagnosis and treatment are essential.^2^ Previously, several modalities of therapy such as retinoids, imiquimod, topical 5-fluorouracil, tacrolimus, laser treatments, phototherapy, surgical interventions, and cryotherapy have been used with a varying degree of success.^3^ In our patient, prior treatments included topical 5-fluorouracil that was discontinued due to erosions and pain, topical imiquimod 5% cream, and multiple sessions of photodynamic therapy without improvement. Recent reports have shown benefit with topical cholesterol 2%/lovastatin 2% treatment in other subtypes of porokeratosis.^4^^,^^5^ We initiated treatment with topical 2% cholesterol/2% lovastatin twice daily. After 2 months of treatment, there was a significant reduction in itch and clinical improvement in erythema with areas of clearing within the center of the lesion.

The cholesterol-mevalonate pathway is fundamental for cell growth and differentiation, cytoskeleton assembly, gene expression, and posttranslational alteration of proteins implicated in intracellular signaling. Cholesterol, an end-product of the mevalonate pathway, is a principal element of the extracellular lipid matrix in the stratum corneum, operating a vital role in implementing and supporting skin barrier capacity.^5^ Mutations in mevalonate synthesis pathway genes have been identified as causative for porokeratosis.^4^ Statins prevent the collection of toxic metabolites by inhibiting HMG CoA reductase and have been used to treat inherited skin disorders with lipid metabolism defects, including porokeratosis.

## Conflicts of interest

None disclosed.
